# Sex Matters during Adolescence: Testosterone-Related Cortical Thickness Maturation Differs between Boys and Girls

**DOI:** 10.1371/journal.pone.0033850

**Published:** 2012-03-29

**Authors:** Jennifer E. Bramen, Jennifer A. Hranilovich, Ronald E. Dahl, Jessie Chen, Carly Rosso, Erika E. Forbes, Ivo D. Dinov, Carol M. Worthman, Elizabeth R. Sowell

**Affiliations:** 1 Developmental Cognitive Neuroimaging Laboratory, Children's Hospital Los Angeles, Los Angeles, California, United States of America; 2 Institute of Human Development, University of California Berkeley Department of Public Health, Berkeley, California, United States of America; 3 Western Psychiatric Institute and Clinic, Department of Psychiatry, University of Pittsburgh, Pittsburgh, Pennsylvania, United States of America; 4 Department of Anthropology, Emory University, Atlanta, Georgia, United States of America; 5 Staglin Center for Cognitive Neuroscience, University of California Los Angeles Department of Psychiatry and Biobehavioral Sciences, Los Angeles, California, United States of America; 6 Laboratory of Neuro Imaging, University of California Los Angeles Department of Neurology, Los Angeles, California, United States of America; 7 Department of Pediatrics, University of Southern California, Los Angeles, California, United States of America; Hangzhou Normal University, China

## Abstract

Age-related changes in cortical thickness have been observed during adolescence, including thinning in frontal and parietal cortices, and thickening in the lateral temporal lobes. Studies have shown sex differences in hormone-related brain maturation when boys and girls are age-matched, however, because girls mature 1–2 years earlier than boys, these sex differences could be confounded by pubertal maturation. To address puberty effects directly, this study assessed sex differences in testosterone-related cortical maturation by studying 85 boys and girls in a narrow age range and matched on sexual maturity. We expected that testosterone-by-sex interactions on cortical thickness would be observed in brain regions known from the animal literature to be high in androgen receptors. We found sex differences in associations between circulating testosterone and thickness in left inferior parietal lobule, middle temporal gyrus, calcarine sulcus, and right lingual gyrus, all regions known to be high in androgen receptors. Visual areas increased with testosterone in boys, but decreased in girls. All other regions were more impacted by testosterone levels in girls than boys. The regional pattern of sex-by-testosterone interactions may have implications for understanding sex differences in behavior and adolescent-onset neuropsychiatric disorders.

## Introduction

Adolescence is a time of dramatic physical, emotional and social change [1] and is also associated with a broad range of problems with behavior and emotion, including sharply increasing rates of depression, suicide, and substance abuse [2]. There is growing interest in achieving a deeper understanding of the normative maturational changes in neural systems that underpin emotion, behavior, and decision-making, in ways that could provide insights into early intervention strategies. One area of interest is focused on understanding puberty-specific maturational changes including the emergence of sex differences (for example the increase in depression in adolescence has been associated directly with the onset of puberty and occurs 2 to 3 times more frequently in girls than boys) [2]. There is evidence for a broader range of sex differences, some established during puberty, that are evident in aspects of cognition and in susceptibility to psychopathology [3], [4], [5]. However, relatively little is known about the neurobiology underlying these differences in maturation. The study of typically developing children and adolescents aids understanding in a period of maturation where the onset of developmental disorders, excessive risk-taking and emphasis on immediate reward can result in pathological outcomes in youth and adults. Models of typical brain development would be improved by evaluation of interactions between hormonal and the still-maturing neural systems responsible for social interactions, affective states and cognitive control. While somewhat controversial, there is significant evidence for sex differences in perceptual (i.e. visuo-spatial) [5], affective (i.e. mood and impulsivity) [6], [7], and language abilities [5] during this period, and these may contribute to differences in practical considerations in, for example, addressing psychopathology and education in our developing children.

Puberty, a sequence of events, which occur during adolescence, is initiated by sex steroid hormone signaling, affecting not only physical sexual maturity, but also reorganization of neural networks that impact behavior. Animal studies have shown that the brain's response to both sensory and steroidal stimulation differs in juveniles, adolescents and adults [8]. Gonadal steroid hormone signaling also has direct functional effects on reproductive behavior through mechanisms akin to neurotransmitter signaling [9]. The long-term organizational effects of hormones on the brain early in development and the activational effects of the same hormones during puberty impact complex behavior [10]. For example, juvenile hamsters castrated prior to and administered testosterone treatments after puberty do not generate normal sexual behavior, highlighting the importance of gonadal steroid hormones in modeling brain circuits [10].

Animal studies have also shown that cellular effects of steroid hormone binding on brain structure are numerous. These include sex differences in the time-course of apoptosis (eg – rat V1) [11], neurotransmitter level changes (eg. cingulate, insular, parietal and occipital cortices, the amygdala, and hypothalamus) [12], [13], and the growth of new cells at puberty [14]. Other sex differences in neurobiology include differences in expression of postsynaptic neurotransmitter receptors [15] or patterns of presynaptic inputs [16], [17] which alter dendritic branching [18], [19], dentritic spine density [20], and connectivity [21]. Further, sex differences in concentrations of both androgen receptors and testosterone (TES) levels in prenatal, peripubertal and adult males and females result in differences in brain structure and function. Focusing on puberty-related sexual differentiation, these studies have found striking sex differences in TES-related patterning in occipital (primary sensory cortex/V1), frontal, and limbic regions. For example, male rodents have more pyramidal branches and dendritic arbors in prefrontal neurons [22] in visual cortices [23] and testosterone levels impact cotrico-cortico motor connections [21]. The higher number of neurons in the visual system in late peripubertal male than female rats has been linked to a suppression of apoptosis by higher testosterone levels in male than female rats [11], [24]. While the animal literature may or may not translate to receptor patterns in humans, we use this as a framework to generate a priori hypotheses in the burgeoning field of relationships between pubertal markers and brain structure in typically developing children and adolescents.

Although the cellular etiology of human brain maturational change observed with structural magnetic resonance imaging (sMRI) is not well understood, human studies have been useful in confirming some of these findings on a macro-scale using non-invasive imaging methods such as sMRI. Recent longitudinal studies with large samples of children and adolescents have shown that gray matter maturation has an inverted u-shaped size-by-age trajectory that varies by region in onset, duration, and shape [25]. As with animals, there are also marked brain changes in humans during puberty and hormone binding in humans likely influences brain development differently in boys than girls. Throughout the brain, there is a net age-related loss of gray matter starting at around age 10 and varying depending on which portion of the cortex measurements are taken, with thinning in dorsal frontal and parietal lobes continuing throughout adolescence and young adulthood that is attributed to gross cellular mechanisms like synaptic pruning and myelination [26], [27], [28], [29]. There is thickening in the medial [30], [31], [32] and lateral temporal lobes until about age 30 [29], [33], [34], perhaps indicative of extended plasticity (i.e., delayed synaptic pruning and myelination) in these regions. The age-related developmental trajectories of male and female adolescents are different [25], and modified by androgen-receptor genetic subtypes [35], confirming results from recent studies of sex-differences in hormone/puberty-correlated maturation ([36], [37], [38]. However, sex differences in both the onset of puberty [39], and in the time-courses of age-related maturation [25], [35], raise questions about whether sex differences in hormone-correlated maturation are due to gross phase differences in development or to actual differences in testosterone-related changes in the brain.

Androgen receptors, targeted by some gonadal steroid hormones, and notably by testosterone, are widely distributed in cortical and subcortical structures [40] and in animals (reviewed above) contribute to significant cellular changes in brain structures during puberty that differ between sexes. In a previous report in the same boys and girls studied here, we found sex differences in volumes of the amygdala and hippocampus, which are important for emotional processing and dense in androgen receptors, that were greater in more sexually mature adolescents. Specifically, sex-differences in the volumes of these specific medial temporal lobe structures were more pronounced in later stages of puberty, where increases in volume were observed in boys, but not girls. We also saw global measures of cortical gray matter volume reductions in later stages of puberty were more prominent in girls than boys [38]. Because frontal lobes are important for impulse control and risk/reward assessments, are known to functionally mature during puberty [41], and have important functional connections with medial temporal lobe structures, such as the amygdala (for review, see [42]), we expected to extend our findings to this report where we focus on cortical thickness.

Using T1-weighted sMRI of 85 adolescent girls and boys matched for pubertal status, assessed using Tanner staging (TS) [43], here, we study sex differences in the relationship between TES levels and cortical gray matter thickness. We did not expect to detect sex differences in cortical thickness, independent of age, in our pubertal status matched population per se, given the small differences in thickness found in previous human studies during the brief age span studied here (approximately 11–14 years) [35]. We did, however, expect to find sex differences in TES-related cortical thickness, independent of age, in regions with high densities of androgen receptors, or high degrees of connectivity with regions expressing high densities of androgen receptors, such as the frontal, limbic, and occipital cortices. Androgen receptors are especially dense within the occipital lobe, limbic cortex (e.g. anterior cingulate gyrus) [44], as well as in numerous non-cortical structures whose projections might exert measurable changes in cortical gray matter thickness [45], [46].

## Results

### Relationships between age and circulating testosterone levels

We tested whether, even within the restricted age range studied, age was associated with TES level. TES levels were positively correlated with age in boys (r = 0.51, p<0.001) but not girls (r = 0.14, p = 0.34).

### Relationship between sex and pubertal status

We tested whether there were differences in pubertal status between participating boys and girls. There was no significant difference between participating boys and girls in Tanner's Stage (p = 0.30).

### Sex Differences

#### Sex differences in cortical thickness and whole brain volume

As expected, we observed no significant differences in our voxel-wise analysis of cortical thickness between boys and girls, independent of age, after correcting for multiple comparisons using the False Discovery Rate (FDR) [47]. While boys had significantly larger (p<0.001) brain volumes than girls (BOYS: mean = 1144518 mm^3^, SD = 112011; GIRLS: mean = 1032897 mm^3^, SD = 79081), mean whole brain cortical thickness was not significantly different (p = 0.28) in participating girls (mean = 2.70 mm, SD = 0.08) and boys (mean = 2.68, SD = 0.09).

#### Sex differences in the effects of TES on cortical thickness, independent of age

To assess whether sex differences in areas with high densities of TES receptors and regions known to be sexually dimorphic during adolescence or adulthood were driven by TES-related change during puberty, we tested for interactions between sex and the effects of TES on gray matter thickness, independent of age using FreeSurfer (http://surfer.nmr.mgh.harvard.edu). We predicted either a less extensive thinning, or even increases in cortical thickness would be associated with increased levels of TES in boys. In girls, conversely, we predicted that more pronounced cortical thinning would generally be associated with increased TES, given findings in longitudinal studies showing a delay in brain maturation (e.g. thinning of frontal cortices) in boys relative to girls [25], [35], and given our previous results of global gray matter volume reductions being more prominent in girls than boys [48].

**Figure 1:**
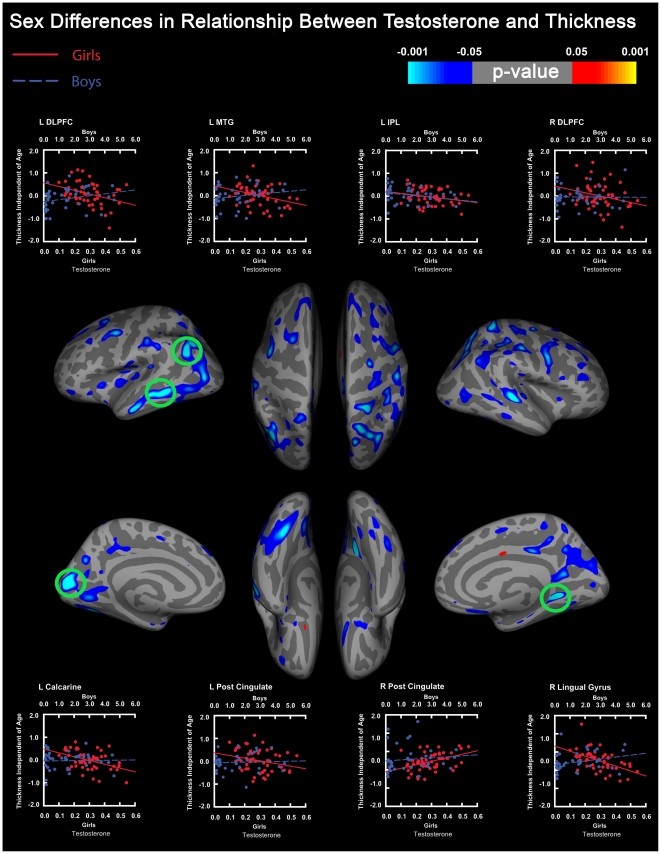
Results from Sex x TES-Thickness interaction. Left side of the figure contains results from the left hemisphere. Green circles indicate regions that survived correction for multiple comparisons using false discovery rate (FDR). Scatterplots represent results extracted from center of significant portion in region indicated. Boys are plotted in blue. Girls are plotted in red. Regions plotted include the left dorsal lateral prefrontal cortex (L DLPFC), middle temporal gyrus (L MTG), inferior parietal lobule (L IPL), calcarine sulcus (L Calcarine), posterior cingulate gyrus (L Post Cingulate), right dorsal lateral prefrontal cortex (R DLPFC), posterior cingulate gyrus (R Post Cingulate), and lingual gyrus (R Lingual Gyrus). Regions surviving FDR include the left inferior parietal lobule, middle temporal gyrus, calcarine sulcus, and right lingual gyrus.

**Figure 2:**
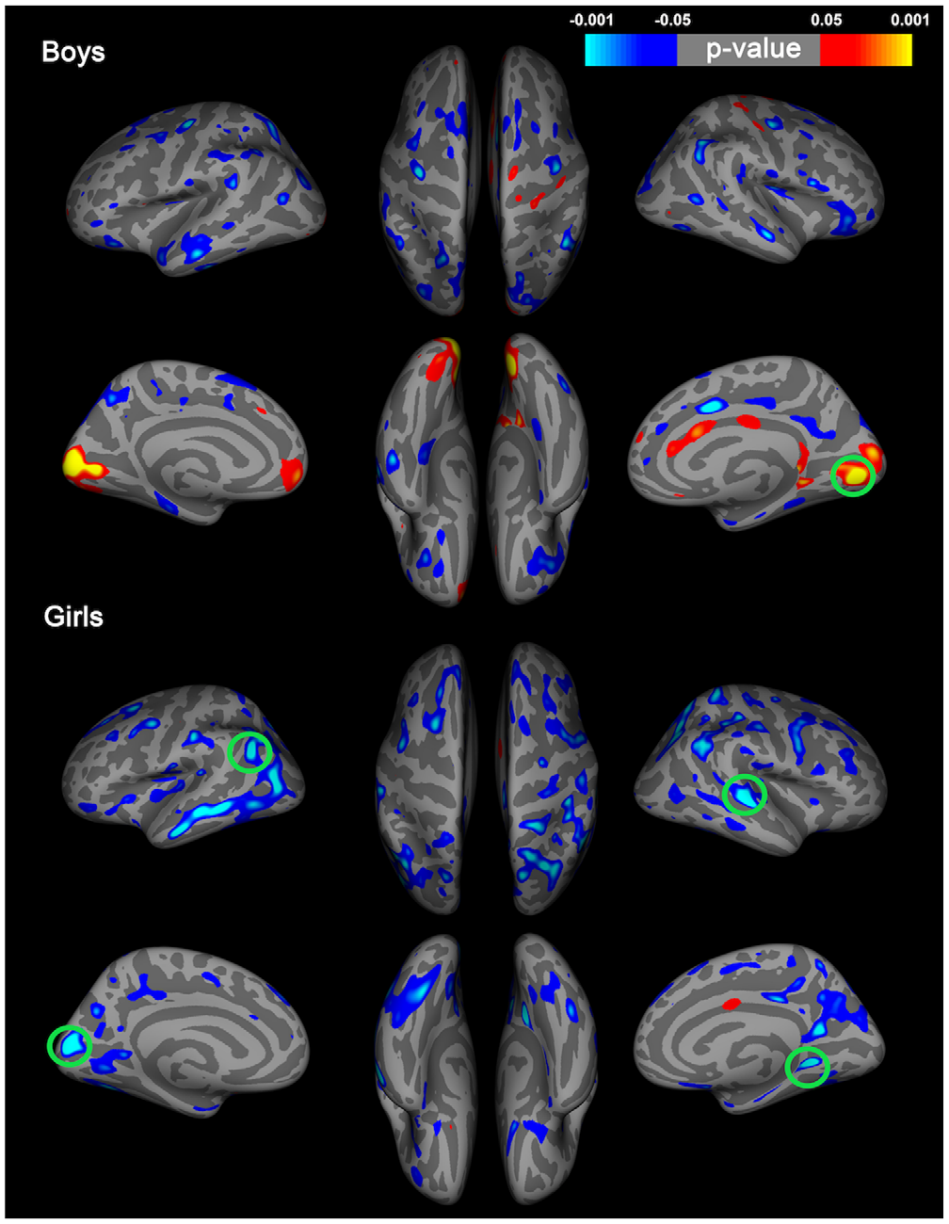
Results from correlating TES levels with thickness, independent of age in boys and girls. Top of figure shows results of analysis in boys. Bottom of figure shows results of analysis in girls. Left side of figure shows results from the left hemisphere. Green circles indicate regions that survive correction for multiple comparisons using false discovery rate (FDR). Regions surviving FDR correction in boys include the right lingual gyrus. Regions surviving FDR correction in girls include the left inferior parietal lobule, calcarine sulcus, right middle temporal gyrus and lingual gyrus.

As predicted, we found sex-by-TES interactions predicting gray matter thickness throughout cortical areas known to be especially dense in ARs, including frontal, limbic, and occipital-visual areas. Regions both specifically predicted and significant, after correcting for multiple comparisons using FDR, were in the occipital lobe (left pericalcarine sulcus; the location of primary visual cortex (V1) and higher order visual regions such as the left cuneus and bilateral lingual gyrus). Interactions were driven by the opposite relationship between TES and gray matter thickness in boys and girls in this occipital lobe region (see Fig. 1). Specifically, higher TES levels were associated with thicker cortex in boys but thinner cortex in girls (see Fig. 2).

We also found sex-by-TES interactions predicting gray matter thickness in the following regions: the bilateral superior and middle frontal gyri, precentral sulcus, posterior cingulate gyrus, lateral orbito-frontal gyrus, left inferior frontal gyrus (including the left pars orbitalis, part of Broca's area), and right medial orbito-frontal gyrus. Here, interactions were driven by multiple mechanisms (see Fig. 1), though generally we found more pronounced TES-related thinning in girls than boys in most regions. In some other regions, interactions were driven by TES related increases in thickness in boys, but not girls (see Fig. 2). While these findings are consistent with our a priori predictions, they did not survive correction for multiple comparisons, and should be interpreted with caution relative to the findings in the occipital lobes.

We also found unpredicted but significant sex-by-TES interactions predicting gray matter thickness, after FDR correction, in the left inferior parietal lobule and left middle temporal gyrus (Fig. 1). Cortical thickness in both girls and boys declined with TES, independent of age, in a similar manner, but the correlation between TES and thickness was significant (after FDR correction) in girls but not boys (Figs. 1 & 2). In the left middle temporal gyrus, TES was associated with cortical thinning in girls, but not boys (Fig. 1).

In addition to the predicted sex-by-TES interactions in occipital cortices, we found unpredicted but significant interactions in other primary sensory areas including the pre- and post- central gyri, locations of primary somatosensory (S1) and motor (M1) regions. The transverse temporal gyrus, the location of primary auditory (A1) cortex showed a trend sex-by-TES-thickness interactions predicting gray matter thickness (α = p<0.05, uncorrected) (Fig. 1). Thickness in the medial occipital cortex (location of primary visual cortex, or V1) and S1 was positively associated with TES levels in boys and negatively associated with TES levels in girls. Thickness in portions of M1 was either not associated, or positively associated with TES levels in boys, but negatively associated with TES levels in girls. A1 appears to be more impacted by TES levels in girls than boys, but in both genders was negatively associated with TES-levels (see scatter plots in Fig. 2).

### Boys

#### Effect of testosterone on cortical thickness, independent of age in boys

We found a positive, significant correlation between TES and the posterior occipital cortex in boys (specifically the right lingual gyrus), corrected for multiple comparisons using FDR (Fig. 2). Other relationships between TES and cortical thickness were observed throughout occipital, limbic and frontal areas, though these regions did not survive correction for multiple comparisons (Fig. 2).

### Girls

#### Effect of testosterone on cortical thickness, independent of age in girls

TES levels were inversely associated with thickness in the occipital lobe (specifically the left calcarine sulcus and right lingual gyrus), independent of age, after correcting for multiple comparisons using FDR (Fig. 2), consistent with our predictions. We also found significant, though not specifically predicted portions of the right superior temporal gyrus to be negatively associated with TES levels after correcting for multiple comparisons using FDR (Fig. 2). Other relationships were found between TES and widespread, bilateral thinning in the posterior cingulate gyrus, entorhinal cortex, precuneus, right parahippocampal gyrus, frontal lobes, including the superior, middle, inferior (including Broca's area) and lateral orbito-frontal gyri, as well as the precentral gyrus of girls, though, these regions did not survive correction for multiple comparisons.

## Discussion

Results from our study are the first to indicate that pubertal hormones impact cortical thickness maturation differently in boys than girls. The current study was unique in using a design in which age and physical sexual-maturity differences are controlled, allowing for the examination of the effects of pubertal maturation independent of age in cortical gray matter thickness. The regional patterns of sex differences in testosterone-related cortical thickening and thinning observed here make sense in light of the animal literature and our own findings [49] that there are sex differences in puberty-related patterning within structures dense in, or highly connected to structures dense in ARs [11], [38], [44], [46], [50]. In general, our results demonstrated that limbic and primary sensory cortices of the occipital lobes increased in thickness with increased testosterone in boys at the same time that testosterone-related decreases in thickness were observed in these regions in girls matched to boys for sexual maturity. Conversely, cortical thinning in frontal cortices appeared more accelerated in girls than boys as testosterone levels increased, though, we were unable to test this hypothesis explicitly given the cross-sectional data.

While we did not predict or detect sex differences in gray matter thickness per se in our sexual maturity-, rather than age-matched population of boys and girls, we did anticipate sex differences in the *pattern of development* of gray matter thickness during pubertal maturation. That is, we observed sex differences in TES-related development of gray matter in our cross sectional sample of adolescents, specifically in cortical regions known to be dense in ARs, like frontal, limbic, and occipital visual areas. To determine what drove sex differences in the association between TES and gray matter, we also tested relationships between TES and gray matter thickness, independent of age, in boys and girls separately. Based on the animal literature, where TES has been found to promote cellular mechanisms that either prevent tissue loss or promote tissue increases at higher levels [11], [23], [51], we expected either increases, or less steep decreases in boys than girls with increasing TES in regions found to be sexually dimorphic in animal models. These hypotheses were supported in our findings of significant sex differences in the TES-related maturational trajectory of visual areas, including early visual areas, specialized in processing the earliest stages of visual processing (e.g. pericalcarine sulcus), and later visual areas (e.g. lingual gyrus and cuneus). Higher TES levels were associated with thinner gray matter in these areas in girls, but with thicker gray matter in boys.

It is important to note that a positive or negative correlation between TES levels and thickness may not represent an effect of TES on thickening or thinning per se. For example, a positive correlation between TES levels and thickness in the visual cortex of boys may represent a preservation of tissue by blocking apoptosis, rather than by stimulating neurogenesis. Indeed, the rodent literature shows that multiple mechanisms are involved in establishing sex differences in the occipital lobe. First, higher TES levels in male than female rats cause an about 20% difference in the number of cells that survive apoptosis [24], leading adult male to have more occipital neurons than adult female rats [11], [23], [51]. There are also differences in synaptic density present in postnatal development, as well as differences in the temporal development of synapses in male and female rats. In early postnatal development, female rats have a higher number of synapses in visual cortex than do male rats, but in later postnatal development, female rats have a decrease in synapse number, while there is evidence that TES spares or even increases these in male rats so that synaptic density becomes similar in fully mature male and female rodents [20].

In addition to predicting sex differences in TES-related patterning in the occipital lobe, we also predicted and found sex differences in frontal cortices including bilateral superior and middle frontal gyri, left inferior frontal gyrus (including the left pars orbitalis, part of Broca's area), bilateral precentral sulcus, and limbic structures (bilateral posterior cingulate gyrus, bilateral lateral orbito-frontal gyrus, right medial orbito-frontal gyrus), though these did not survive correction for multiple comparisons. In general, we found more pronounced TES-related thinning in the frontal regions of girls than boys. These differences may relate to sex differences in adolescent cognition and behavior. For example, differences in puberty related patterning in Broca's area may relate to findings that adolescent girls on average out perform boys in some language tasks [5], or explain why these differences disappear in adulthood (for review, see [52]). Similarly, the less prominent or delayed puberty-related frontal lobe development in boys may explain sex differences in the developmental trajectories found in adolescents, such as the initial delay and subsequent acceleration in male frontal lobe thinning relative to females [27], [35], which has been proposed as an explanation of the peripubertal male bias in accidents, violence, and psychopathology such as drug abuse and suicide [35]. While these (uncorrected) findings should be interpreted with more caution, they may be useful in generating further hypotheses for future studies.

Though unpredicted at the outset of this study, we also found significant (with correction for multiple comparisons using FDR) sex differences in TES-related patterning in the left inferior parietal lobe and left middle temporal gyrus. Our findings may be consistent with recent reports of sex differences in longitudinal developmental trajectories as well as evidence that variability in the maturation of these structures is accounted for by AR subtype. More efficient TES binding was associated with more “masculine” patterning in this region in a previous report [35]. Further, longitudinal studies have shown that during puberty, the temporal lobes mature/thicken more rapidly in boys than girls [35]. Here we show additional evidence that these findings could be rooted in sex differences in TES-related cortical patterning.

Interestingly, we found that TES-levels were inversely associated with thickness in the lateral temporal lobe in girls (corrected for multiple comparisons using FDR), though this region is known to thicken with age during adolescence [33], suggesting that TES is on average suppressing mechanisms that cause apparent thickening on MRI. This is not consistent with findings, specifically in girls, that more efficient TES binding, based on AR receptor subtype, is associated with more rapid thickening [35]. It is possible that a longitudinal study could shed light on these complex interactions. It is also possible that differences in TES levels, age, or AR receptor subtype composition between our population of girls and participants in other studies explain this discrepancy.

In contrast to animal studies that can experimentally control TES levels, this is a study of typically developing youth, which must rely on correlations between TES and thickness. While correlation is not causality, we believe our findings in conjunction with evidence from experimental studies in the animal literature support the hypothesis that changes in TES levels directly impact changes in human brain structure. Given the cross-sectional design of our study, future longitudinal studies are needed to better disentangle how age, time, and TES levels (and perhaps specific experiences) modulate changing brain structure. While our measures of testosterone were collected in the morning, variations in testosterone collected at different times in the day should be the subject of future study. The current study represents an important contribution to the literature on adolescent brain development providing new insights into the importance and specificity of sex-specific changes in neurodevelopment during pubertal maturation. Given the broad range of behavioral and emotional health problems that emerge at this point in development—including sex differences in depression, substance use and some risk-taking behavior, this work highlights the need for further investigations to further delineate sex and puberty specific changes during this dynamic and important period of brain development.

## Materials and Methods

### Subjects

130 healthy adolescents from Pittsburgh, PA and the surrounding community were recruited through advertisements, flyers, and demographically targeted phone lists. Adolescents were free of lifetime psychiatric disorders, did not have braces, and had no history of head injury, serious medical illness, or psychotropic medication. The University of Pittsburg Institutional Review Board approved human participants research. Written informed consent was obtained from a parent or legal guardian, verbal assent was obtained from the adolescent participants, and all clinical investigation was conducted according to the principles expressed in the Declaration of Helsinki. Twenty-nine participants (22%) were of African-American, two (1.5%) Asian, and two Native-American (1.5%) decent. The remaining participants were Caucasian (75%). Three participants withdrew, seven were excluded due to inadequate image quality, and twenty-eight participants were excluded because we were unable to assay circulating TES levels. A subset of the same subjects described in this report have been evaluated to assess relationships between pubertal maturation and brain activation [53] and to assess relationships between pubertal development and structures in the medial temporal lobes (MTL) [38]. In this manuscript, we do not re-evaluate changes in the MTL. While most participants are used in both studies, some had to be excluded due to issues related to image quality in the MTL, which is more susceptible to artifacts. Table 1 provides demographic descriptions of the 85 participants.

**Table 1:** Demographic characteristics of 85 normally developing adolescents, by sex.

Demographics			
*Sex*	*Description*	Mean	SD
**Girls**	Age	11.93	0.64
	Age Range	10.75–13.48	--
	Number (N)	49	--
	Mean TS	3.40	1.63
	TS Range	2–5	--
	Mean Testosterone	0.29	0.11
	Testosterone Range	0.10–0.54	--
	Handedness (Number Right)	45	--
**Boys**	Age	12.88	0.67
	Age Range	11.73–13.99	--
	Number (N)	36	--
	Mean TS	3.92	2.37
	TS Range	1–4	--
	Mean Testosterone	1.52	1.37
	Testosterone Range	0.12–5.43	--
	Handedness (Number Right)	33	--

*Sex Differences*	
	p-value
Age	0.00*
TS	0.30
Testosterone	0.00*

Demographics from 85 participating adolescent boys and girls. Demographics are tabulated for girls (TOP) and boys (MIDDLE) as data from each sex was analyzed separately in some statistical tests. Sex differences (BOTTOM) in key demographics of participating boys and girls are tabulated. A one-tailed, two-independent sample t-test was used to calculate sex differences in TS. Two-tailed, two-independent sample t-tests were used to calculate sex differences in age and circulating testosterone.

* denotes significance (p<0.05).

### Sexual maturity

Participants underwent a physical examination by a research-trained nurse practitioner to determine stage of sexual maturation with the criteria specified by Marshall and Tanner [43]. Group descriptions are detailed in Table 1. Boys and girls were matched for TS using group means.

### Circulating testosterone levels

Blood samples were collected and analyzed for TES level in both boys and girls. Samples were obtained at the same time for all subjects (between 8:20 and 8:35 AM), using minimally invasive finger-stick procedure developed by Worthman and colleagues [54]. This method provides several advantages over salivary assays for gonadal steroids, and the correspondence of bloodspot-derived level and plasma level is high. Hormone assays were a modification of a commercially available serum/plasma radioimmunoassay kit (T: DSL/Beckman Coulter, Miami FL; E2 Siemens, Los Angeles, CA). Sensitivity measured as the minimum detectable dose (MDD) and inter-assay coefficients of variation (CV) for low, medium and high BioRad external controls for TES were MDD = .04 ng/mL; CV = 7.2% (low), 11.4% (medium) and 4.3% (high). This assay technique is sensitive enough to detect testosterone levels in girls, who have much lower levels than boys.

### Structural image acquisition

All subjects were scanned in a Siemens Allegra head-only 3-Tesla magnet with a 3D T1-weighted protocol (Siemens, Malvern, PA). Scan parameters were as follows: repetition time (TR), 1540 ms; echo time (TE), 3.04 ms, flip angle, 8°; field of view (FOV) 256×256; image voxel size, 1×1×1 mm; acquisition time, 4.48 m.

### Image processing

Preprocessing on structural images were conducted in the UCLA Laboratory of Neuro Imaging (LONI) Pipeline Processing Environment [55], [56] and using FreeSurfer's automated segmentation software (FreeSurfer 4.0.3, http://surfer.nmr.mgh.harvard.edu), as described in the work of [57], [58], [59]. During preprocessing, T1-weighted images for each participant were motion corrected using a hybrid watershed/surface deformation procedure [60], brain extracted, intensity normalization [61] tessellation of the gray matter white matter boundary, automated topology correction [62], [63], and surface deformation following intensity gradients to optimally place the gray/white and gray/cerebrospinal fluid borders at the location where the greatest shift in intensity defines the transition to the other tissue class [58], [64].

Additional data processing and analysis was conducted outside of the LONI Pipeline environments, including surface inflation [57], registration to a spherical atlas, which utilized individual cortical folding patterns to match cortical geometry across subjects [65]. This method uses both intensity and continuity information from the entire three dimensional MR volume in segmentation and deformation procedures to produce representations of cortical thickness, calculated as the closest distance from the gray/white boundary to the gray/CSF boundary at each vertex on the tessellated surface [64]. The maps were created using spatial intensity gradients across tissue classes and are therefore not simply reliant on absolute signal intensity. The maps produced are not restricted to the voxel resolution of the original data and thus are capable of detecting submillimeter differences between groups. Procedures for the measurement of cortical thickness have been validated against histological analysis [66] and manual measurements [67], [68]. Freesurfer morphometric procedures have been demonstrated to show good test-retest reliability across scanner manufacturers and across field strengths [69]. It should be noted, that while we studied the *volumes* of the amygdala and hippocampus in our previous report [38], as both are treated as 3-dimensional volumetric shapes, rather than as part of the 2-dimensional cortical ribbon, and we did not investigate those regions in this analysis of cortical thickness.

### Statistical Analyses

We used a one-tailed two-independent sample t-test [70] to test whether participating boys had larger whole brain volumes than participating girls. Sex differences in whole brain thickness were evaluated using a two-tailed two-independent sample t-test [71]. Mean thickness was calculated using regional measures extracted using Freesurfer tools and by averaging thickness in each region in both hemispheres. We also used a two-tailed two-independent sample t-test to test whether participating boys and girls were in significantly different stages of puberty, based on Tanner's Stage.

All other analyses were conducted within the FreeSurfer statistical software package using an alpha of p<0.05 (both voxel-wise and when correcting for multiple comparisons using FDR) and simultaneous multiple regression analysis. To assess SEX-by-TES-thickness interactions, independent of age, we used the following linear model: THICKNESS = CONSTANT+*beta_1_*SEX+*beta_2_*TES+*beta_3_*SEX+TES *beta_4_*AGE+ERROR. To understand the underlying effects of TES on thickness in boys and girls separately, independent of age, we used the following linear model: THICKNESS = CONSTANT+*beta_1_*TES+*beta_2_*AGE+ERROR.
